# A Comparative Study of Two Different Polarity Extracts of *Angelica sinensis* on Growth, Body Composition, and Metabolism in Juvenile Carp

**DOI:** 10.1155/anu/8840979

**Published:** 2026-02-11

**Authors:** Huatao Li, Haijing Liu, Guichuan Chen, Wang Xiong, Boyan Li, Qihui Yang, Jing Xu, Gangfu Chen

**Affiliations:** ^1^ Fishes Conservation and Utilization in the Upper Reaches of the Yangtze River Key Laboratory of Sichuan Province, Neijiang Normal University, Neijiang, 641100, Sichuan, China, njtc.edu.cn; ^2^ College of Fisheries, Neijiang Normal University, Neijiang, 641100, Sichuan, China, njtc.edu.cn; ^3^ College of Fisheries, Guangdong Ocean University, Zhanjiang, 524088, Guangdong, China, gdou.edu.cn

**Keywords:** *Angelica sinensis* extracts, body composition, carp, growth, metabolism

## Abstract

This study investigated the effects of low and high polarity extracts (LPE and HPE) of *Angelica sinensis* (AS) on growth, body composition, and metabolism in carp (*Cyprinus carpio* var. Jian). Over a 42‐day period, 780 fish were randomly separated into 13 groups with three replicate aquariums respectively. Thirteen groups received the feeding of basic diet, six LPE diets, and six HPE diets, respectively. The results indicated that dietary LPE decreased weight gain (WG), condition factor (CF), the activities of Na^+^, K^+^‐ATPase, γ‐glutamyl transpeptidase (γ‐GT), trypsin and lipase in digestive organs, plasma total amino acids (TAAs), triglyceride (TG) and ammonia levels, lipid productive value (LPV), and ammonia excretion rate (AER; *p* < 0.05), increased the activities of alkaline phosphatase (AKP), glutamate‐oxaloacetate transaminase (GOT), glutamate‐pyruvate transaminase (GPT), and α‐amylase in digestive organs, the content of total protein (TP) and glucose (GLU) in plasma, protein productive value (PPV), oxygen consumption rate (OCR), and O:N ratio in carp (*p* < 0.05). At the same time, dietary HPE increased WG, CF, the activities of lactate dehydrogenase (LDH), trypsin and lipase in digestive organs, the content of TP in plasma and LPV, PPV, OCR, and O:N ratio (*p* < 0.05) and decreased the activities of GOT and GPT in hepatopancreas as well as the content of ammonia, TAA, and GLU in plasma of carp (*p* < 0.05). According to the above findings, dietary LPE inhibits the growth and accumulation of body lipid and enhances the accumulation of body proteins by decreasing the digestion and absorption of lipids as well as amino acid catabolism, and increasing the catabolism of sugar and fat in fish. Dietary HPE enhances the growth and accumulation of body lipid and proteins by decreasing the catabolism of amino acid and increasing the digestion and absorption of proteins and lipids and the catabolism of sugar in fish.

## 1. Introduction

*Angelica sinensis* (AS) has a long history of use and is one of the most widely demanded and cultivated medicinal plants in China [1]. Modern pharmaceutical researches show the various pharmacological activities of AS having antioxidative, anti‐inflammatory, antivirus, anti‐fatigue, immune support, and hematopoiesis function in mammals [2, 3]. During the harvesting and processing of AS, it needs to go through various stages such as mining, drying, stacking, tying, combing, shelving, baking, and slicing [4]. During this process, a large amount of AS by‐products such as damaged roots, broken roots, lateral roots, whiskers, and miscellaneous pieces are produced [4]. In the current list of feed raw materials in China, AS, its specific parts, and its crude extracts belong to feed raw materials [5]. However, the application of AS in aquafeeds remains limited, potentially due to existing controversy regarding its effects on fish growth performance. The growth‐promoting effect of AS in *Carassius auratus* has been noted in previous investigation [4]. However, other report indicated that AS suppresses body weight (BW) gain in *Hucho taimen* and *Acipenser schrenckii* [6, 7]. Although showing a trend toward increased weight gain (WG) in *Pelteobagrus vachelli*, oral administration of AS did not produce a statistically significant difference relative to controls [8]. Little is known about the reason of effect of AS on the growth in aquatic animals.

The effect of AS on fish growth may be associated with its constituents. The main active constituents of AS are ligustilide, ferulic acid, polysaccharides, and so forth [9]. It has been reported that AS suppresses body WG in obese mice [10]. Ligustilide (low polarity component in AS) alleviates the lipid accumulation in rats with diabetes mellitus [11]. AS high polarity extract (HPE) containing polysaccharides enhances the WG in carp [12]. Therefore, it is reasonable to hypothesize that AS low polarity component cut down the effect of its high polarity component on the growth, which leads to the weakness of growth promoting function of AS in fish. Nevertheless, the influence of AS low polarity extract (LPE) containing ligustilide on piscine growth performance remains poorly understood. The growth of fish is closely related to their digestion, absorption, and metabolism [13]. Previous studies in our laboratory showed that dietary HPE improves the digestive and absorptive abilities in carp [12]. Glutamate‐oxaloacetate transaminase (GOT) and glutamate‐pyruvate transaminase (GPT) play the important roles in protein metabolism in fish [14]. Ammonia is the final product of protein metabolism in fish [15]. Studies have demonstrated that dietary AS medium polarity extract (MPE) increased the activities of GOT and GPT in hepatopancreas and muscles and decreased the level of ammonia in plasma in carp [16]. Lactate dehydrogenase (LDH) acts as an essential enzyme in glycolysis, facilitates energy production [17]. It has been reported that dietary MPE containing ferulic acid suppressed the trichlorfon‐induced decrease in LDH activity in muscles of Crucian carp [18]. Dietary AS polysaccharide has been shown to change the content of protein and fat in fish body as well as of glucose (GLU) and lipids in blood of golden pompano [19]. The changes in fish body composition and energy substances in blood are closely related to their metabolism as well as gastrointestinal function [13]. However, limited data are available regarding on how LPE make effect on the digestion, absorption, body composition, and metabolism and on how HPE make effect on body composition in fish.

The gastrointestinal function and metabolism in carp (*Cyprinus carpio* var. Jian) received the probing process here. This work aimed at comparing the way how LPE and HPE influences quantifiable growth metrics and biochemical body composition profiles in fish. The research outcome may reveal the mechanisms why supplementing AS affects fish growth and provide a basis for its application as a functional raw material in fish feed.

## 2. Materials and Methods

### 2.1. Animal Ethics Statement

Ethical approval for the experimental procedures was obtained from the ethics committee of Neijiang Normal University (Grant Number JM2021‐15). The author has complied with all relevant ethical laws.

### 2.2. Chemicals

All solvents and chemicals used were of analytical grade unless otherwise specified. Petroleum ether, ethyl acetate, and ethanol were obtained from Chengdu Kelong Chemical Reagent Factory (Chengdu, Sichuan, China). High‐purity reference standards were sourced as follows: ligustilide (≥98% purity) from Chengdu Herbpurify Co., Ltd. (Chengdu, Sichuan, China); quercetin and ferulic acid (both ≥99% purity) from Shanghai EkearBiotech Co., Ltd. (Shanghai, China); catechin and kaempferol (both ≥99% purity) from Sigma–Aldrich Co., LLC (St. Louis, MO, USA). Analytical grade chemicals were used for all other reagents.

### 2.3. AS Extracts Preparation

The by‐products of AS (containing 5.27% heads, 18.18% coarse tails, 57.04% fine tails, and 8.95% beards of roots [4]) were purchased from Huichuan Shihong Traditional Chinese Medicine Professional Cooperative in Weiyuan County, Gansu, China. The Neijiang Normal University carried out botanical recognition. AS root powder was sequentially extracted using solvents of increasing polarity, following the protocol established by the literature [16]. The extraction series included: (1) petroleum ether (low polarity), (2) ethyl acetate (medium polarity), (3) ethanol, and (4) water (high polarity). This gradient extraction approach yielded corresponding fractions for subsequent analysis. The petroleum ether extract is LPE. The aqueous extract is HPE. After extraction, the dry extracts received the maintenance in the dark air tight bottle and the storage at −80°C for subsequent application.

### 2.4. The Composition Analyses of AS Extracts

The composition analyses of LPE and HPE was implemented using High performance liquid chromatography (HPLC) following the explanation by the literature [18]. Two fresh and saturated solution were in preparation by the dissolution of LPE and HPE in methanol (grade of chromatography), respectively. The saturated solutions were respectively transferred into HPLC for ligustilide, ferulic acid, catechin, quercetin, as well as kaempferol quantitative analysis. All extractions and analyses were performed in triplicate (*n* = 3) to ensure methodological reproducibility. The content of components above in LPE and HPE is shown in Figure 1.

Figure 1The investigations on the compositions of low (a) and high (b) polarity extracts of *Angelica sinensis* using High performance liquid chromatography (HPLC). The present experimental process received the repetition three times, and consistent outcomes were gained.

**Figure:**
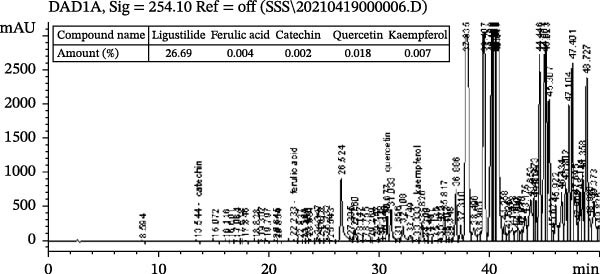
(a)

**Figure:**
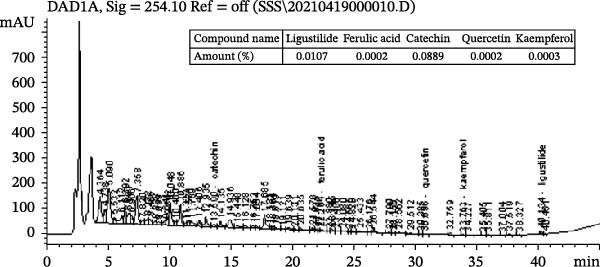
(b)

The proximate composition of HPE, including moisture, crude protein, crude lipid, and ash contents, was determined following standard AOAC methods [20, 21]. Carbohydrate analysis was performed according to the literature [22]. Polysaccharide content calculated as: polysaccharide = total sugar − reducing sugar. The chemical components of HPE, including moisture, crude protein, crude lipid, ash, and various sugar fractions, were summarized in Table 1.

**Table 1:** Composition of high polarity extract of *Angelica sinensis* (HPE).

Ingredients	Content (%)
Moisture	7.55 ± 0.30
Crude protein	19.55 ± 1.18
Crude lipid	0.17 ± 0.01
Ash	4.96 ± 0.30
Total sugar	70.28 ± 2.08
Reducing sugar	20.24 ± 1.50
Polysaccharide	50.84 ± 0.76

*Note*: Values are means ± SD of 3 replicates.

### 2.5. Animal Experimentation

#### 2.5.1. Diets of Experimentation

In this study, a total of 13 experiment diets including one basal diet, six LPE diets, and six HPE diets received the formulation and production following the explanation previously [23]. The crude lipid and crude protein contents of the basal diet were 5.18% and 34.32%, respectively. LPE and HPE, respectively, received the addition into the basic diet for providing 0.0 (control), 2.0, 4.0, 6.0, 8.0, 10.0, and 12.0 g kg^−1^ diet. To accommodate the supplementation of LPE and HPE, the proportion of wheat flour was reduced. The pellet which feeds for every treatment was dried in the oven at 50°C for 24 h, finally sealed in sample bags and store at −20°C until use. Proximate analysis were conducted with a FOSS DA1650 Near Infrared Reflectance Spectrometer (NIRS, Hillerød, Denmark) for moisture, crude protein, crude lipid, and ash investigations of diets, as recommended by the literature [24, 25]. Table 2 presents the basal diet of formulation.

**Table 2:** Composition and proximate analysis on the basal diet.

Ingredients	%	Proximate analysis	%
Fish meal	14.00	Dry matter	93.35
Meat bone meal	7.00	Crude protein	34.32
Peanut meal	28.00	Crude lipid	5.18
Sunflower seed meal	11.00	Crude ash	8.11
Rapeseed meal	6.00	—	—
Wheat flour	27.60	—	—
DL‐methionine	1.00	—	—
Threonine	0.70	—	—
Lysine	0.50	—	—
Fish oil	1.10	—	—
Corn oil	1.10	—	—
Vitamin mixture^a^	1.00	—	—
Mineral mixture^b^	1.00	—	—

^a^Presented the per kg of vitamin mix below: folic acid (96%), 0.52 g; D‐biotin (2%), 5.00 g; meso‐inositol (99%), 52.33 g; D‐calcium pantothenate (90%), 2.73 g; niacin (99%), 2.82 g; cyanocobalamin (1%), 0.10 g; ascorhyl acetate (93%), 7.16 g; riboflavine (80%), 0.63 g; pyridoxine HCl (81%), 0.92 g; menadione (23%), 0.43 g; thiamin nitrate (90%), 0.11 g; cholecalciferol (500,000 IUg^−1^), 0.48 g; DL‐α‐tocopherol acetate (50%), 20.00 g; retinyl acetate (500,000 IUg^−1^), 0.80 g.

^b^Presented the per kg of mineral mix below: CaCO_3_, 897.98 g; KI (4% I), 2.90 g; ZnSO_4_·7H_2_O (23% Zn), 21.64 g; MnSO_4_·H_2_O (32% Mn), 4.09 g; Na_2_SeO_3_·5H_2_O (1% Se), 2.50 g; CuSO_4_·5H_2_O (25% Cu), 1.20 g; FeSO_4_·7H_2_O (20% Fe), 69.70 g.

#### 2.5.2. Feeding Trial

Carps used in this experiment were all collected from a cultured population maintained a facility in Neijiang, Sichuan, China. Prior to experimentation, fish underwent a 15‐day acclimation period in controlled laboratory environments according to standardized procedures [23]. The index values for dissolved oxygen (DO), pH, ammonium‐nitrogen, and nitrite were complied with the criteria outlined in the literature [22]. The assessment of water quality parameters followed the procedure outlined by the literature [22]. After acclimated to the laboratory conditions for 15 days, 780 fish exhibiting the original weight of 5.91 ± 0.08 g on average were randomly separated into 13 groups with three replicate aquariums, respectively. The respective aquarium exhibited the size of 55 cm × 32 cm × 40 cm, covering 20 fish. Thirteen groups received the 42‐day feeding of basic diet (control group), six LPE diets (containing 2.0, 4.0, 6.0, 8.0, 10.0, and 12.0 g LPE kg^−1^ diet, respectively), as well as six HPE diets (containing 2.0, 4.0, 6.0, 8.0, 10.0, and 12.0 g HPE kg^−1^ diet, respectively). All fish in the respective group received the four‐time feeding on a day‐to‐day basis and the rigorous observation for ensuring them satiated with no overfeeding in 42 days. Uneaten feed particles were counted and their weight was calculated (weight of uneaten feed particles = number of uneaten particles × average weight of each particle) and used for correction of feed intake (FI).

At the end of the rearing experiment, fish in each aquarium were sequentially counted and weighed. The initial number, final number, initial BW (IBW), final BW (FBW), and feed consumed were used to calculate the survival rate (SR) [26], WG, specific growing rate (SGR), FI [27], feed efficiency (FE), and protein efficiency ratio (PER) [20].

Survival rate SR, %=100×final fish count/initial fish count.

WG g fish−1=FBW g fish−1−IBW g fish−1.

SGR % day−1=100×ln FBW− ln IBW/experimental duration day.

FI g fish−1=Feed offered g− feed residues g/final number of fish.

FE %=100×WG/FI.

PER=WG/FI×dietary protein content.

At the commencement and conclusion of the feeding trial, body composition samples were collected as follows: Initial sampling: 30 fish were randomly selected from the source population. Final sampling: Five fish were randomly sampled in per aquarium. Body moisture content (BMC) was determined by weight loss after drying at 105°C. Subsequently, dried samples were homogenized and then analyzed via the NIRS to quantify body protein, body lipid, and body ash content (BPC, BLC, and BAC). These measurements facilitated calculation of protein productive value (PPV), lipid productive value (LPV), and ash productive value (APV) according to the literature [28].

When the experiment for feeding was completed, 10 fish in per aquarium were anesthetized in water containing ethyl carbamate and then kept in ice for BW and ruler measurement for calculation of condition factor (CF) [28]. Blood was collected from fish in each group via caudal puncture using heparinized syringes. The plasma and red blood corpuscles received the isolation through centrifugation for 15 min at 1000 × *g* at 4°C in 1 h in blood. Blood plasma was collected for determination of enzyme activities including GOT and LDH, and biochemical parameters including triglyceride (TG), GLU, total protein (TP), total amino acid (TAA), and plasma ammonia (PA).

Subsequently, hepatopancreas and intestinal tissues were immediately excised. Organ weights (for calculation of hepatosomatic index [HSI] and intestosomatic index [ISI]) [16] and intestinal length (for calculation of intestine length index [ILI]) [28] were measured. All tissues were then snap‐frozen in liquid nitrogen and stored at −80°C for subsequent analysis.

PPV %=100×FBW×final BPC−IBW×initial BPC/FI×dietary protein content.

LPV %=100×FBW×final BLC−IBW×initial BLC/FI×dietary lipid content.

APV %=100×FBW×final BAC−IBW×initial BAC/FI×dietary ash content.

CF %=100×wet body weight g fish−1/wet body length cm3.

HSI %=100×wet hepatopancreas weight g fish−1/wet body weight.

ISI %=100×wet intestine weight g fish−1/wet body weight.

ILI %=100×wet intestine length cm/wet body length cm.

Tissue samples were thoroughly homogenized with nine volumes (*w*/*v*) of ice‐cold physiological saline. The homogenates were then centrifuged at 3200 × *g* for 20 min at 4°C, and the resulting supernatant were used for the anzymatic assays of trypsin, lipase, α‐amylase, Na^+^, K^+^‐ATPase, alkaline phosphatase (AKP), γ‐glutamyl transpeptidase (γ‐GT), GOT, GPT, and LDH activities and protein contents.

#### 2.5.3. Metabolic Experiments

After feeding trial ended, experimenters collected five individuals from each aquarium and weighed (BW, g) for metabolic experiments. The oxygen consumption rate (OCR, mg g^−1^ h^−1^) and ammonia excretion rate (AER, mg g^−1^ h^−1^) were evaluated following the methodology described by the literature [14], with only minor adjustments. Prior to the experiment, 100 L of fresh water was aerated for 24 h in aquaria using an aerator (SB‐748) to equilibrated to room temperature (22°C) and achieve saturation with DO. The initial DO (IDO, mg L^−1^) and initial ammonia concentrations (IACs, mg L^−1^) in the water were measured using a Leici JPBJ‐608 DO analyzer and Jiancheng detection kits (Nanjing, Jiangsu, China), respectively. For the experiment, approximately 50 times the BW of the fish in saturated DO water (WV, L) was carefully transferred into polyethylene terephthalate (PET) bottles (4.5 L), which five individuals were then paced into. Excess air was expelled from the bottle, which was immediately sealed, and timing was initiated, with the experiment lasting for a defined duration (DT) of 0.5 h. Once the experiment was finished, the final DO (FDO, mg L^−1^) and final ammonia concentrations (FACs, mg L^−1^) in water were measured immediately again. FDO was maintained above 5.0 mg L^−1^ throughout to prevent physiological stress. Finally, the OCR and AER were calculated per hour and per gram of fish body mass according to the following formula. Based on OCR and AER, the O:N ratio is calculated using the following formula. All treatment groups underwent triplication.

OCR=IDO−FDO× WV/0.5×BW.

AER=FAC−IAC× WV/0.5×BW.

O:N ratio=14×OCR/16×AER.

#### 2.5.4. Biochemical Analysis

Following the report by the literature [18], the activity of GPT and LDH and the content of PA received the measurement. The content of TG and TC and the activity of GOT were determined with the approach according to the literature [11]. GLU, TP, and TAA concentrations were determined using standardized commercial assay kits (Nanjing Jiancheng Bioengineering Institute, China) in strict accordance with the manufacturer’s operating procedures. The activities of Na^+^, K^+^‐ATPase, AKP, and γ‐GT were determined according to the method described by the literature [28]. The assays of trypsin, lipase, and α‐amylase activities were performed using the techniques established by the literature [16]. Protein content was quantified following the reported procedure [29].

### 2.6. Statistical Analysis

All data are subjected to one‐way analysis of variance (ANOVA) following the method of the literature [27], with results expressed as mean ± standard deviation (SD). Based on multiple range assays of Duncan [30], this study found noticeable differences. A probability level of 0.05 is set as the criterion for statistical significance. Based on SPSS 26.0 for Windows software, the statistics is analyzed.

## 3. Results

### 3.1. How Dietary LPE and HPE Influences Fish Growth Performance

**Figure 2:**
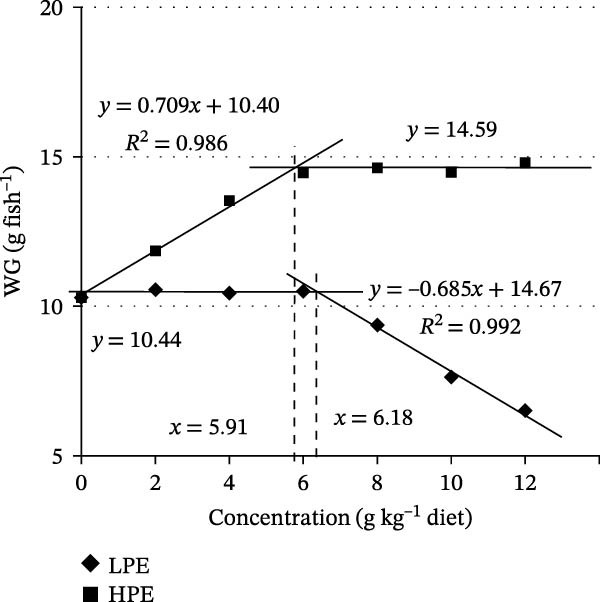
Broken‐line analysis of weight gain (WG) for Jian carp fed diets containing graded levels of the low or high polarity extract of *Angelica sinensis* (LPE or HPE) for 42 days. The optimal LPE and HPE requirement of juvenile Jian carp was 6.18 and 5.91 g kg^−1^ diet, respectively. The data represent the means of 3 replicates, with 20 fish in each replicate.

The effects of dietary supplementation with graded levels of LPE and HPE on growth performance of Jian carp are presented in Table 3. Compared with participants not receiving the treatment, both LPE and HPE supplementation significantly modulated growth parameters, with LPE reducing and HPE enhancing the FBW, WG, SGR, FI, FE, and PER (*p* < 0.05, Table 3). There was no recorded mortality among the experimental subjects (Table 3). The highest values of these parameters were observed in fish fed the diet containing 6.0 g HPE kg^−1^, whereas a decline was noted at 8.0 g LPE kg^−1^ supplementation (Table 3). Broken‐line regression analysis indicated that the optimal dietary levels of LPE and HPE for WG were determined to be 6.18 and 5.91 g kg^−1^, respectively (Figure 2).

**Table 3:** Initial body weight (IBW), final body weight (FBW), weight gain (WG), specific growth rate (SGR), feed intake (FI), feed efficiency (FE), protein efficiency ratio (PER), and survival ratio (SR) of Jian carp fed diets containing graded levels of the low or high polarity extract of *Angelica sinensis* (LPE or HPE) for 42 days.

Concentration (g kg^−1^ diet)	IBW (g fish^−1^)		FBW (g fish^−1^)		WG (g fish^−1^)		SGR (% day^−1^)	
	LPE	HPE	LPE	HPE	LPE	HPE	LPE	HPE
0.0	5.91 ± 0.08^aa’^		16.2 ± 0.69^ba”^		10.29 ± 0.61^ba^		2.4 ± 0.07^ba’^	
2.0	5.90 ± 0.09^a^	5.90 ± 0.05^a’^	16.45 ± 0.85^b^	17.75 ± 1.10^a’b’^	10.55 ± 0.93^b^	11.85 ± 1.13^a’b’^	2.44 ± 0.15^b^	2.62 ± 0.16^a’b’^
4.0	5.93 ± 0.08^a^	5.92 ± 0.10^a’^	16.37 ± 0.88^b^	19.45 ± 0.90^b’c’^	10.43 ± 0.85^b^	13.53 ± 0.99^b’c’^	2.41 ± 0.12^b^	2.83 ± 0.15^b’c’^
6.0	5.90 ± 0.09^a^	5.88 ± 0.08^a’^	16.40 ± 1.03^b^	20.33 ± 1.18^c’^	10.50 ± 0.97^b^	14.46 ± 1.10^c’^	2.43 ± 0.12^b^	2.95 ± 0.11^c’^
8.0	5.89 ± 0.06^a^	5.89 ± 0.08^a’^	15.27 ± 1.06^b^	20.52 ± 1.51^c’^	9.38 ± 1.00^b^	14.63 ± 1.53^c’^	2.26 ± 0.14^b^	2.97 ± 0.19^c’^
10.0	5.92 ± 0.08^a^	5.93 ± 0.07^a’^	13.55 ± 0.77^a^	20.40 ± 1.06^c’^	7.63 ± 0.69^a^	14.48 ± 1.00^c’^	1.97 ± 0.10^a^	2.94 ± 0.10^c’^
12.0	5.91 ± 0.09^a^	5.91 ± 0.06^a’^	12.42 ± 0.68^a^	20.70 ± 1.70^c’^	6.51 ± 0.62^a^	14.79 ± 1.67^c’^	1.77 ± 0.11^a^	2.98 ± 0.19^c’^

	**FI (g fish^−1^)**		**FE (%)**		**PER**		**SR (%)**	
	**LPE**	**HPE**	**LPE**	**HPE**	**LPE**	**HPE**	**LPE**	**HPE**

0.0	20.54 ± 0.56^ca’^		50.09 ± 2.01^ca’^		1.46 ± 0.06^ca’^		100.00 ± 0.00^aa’^	
2.0	20.94 ± 0.45^c^	21.27 ± 0.49^a’b’^	50.35 ± 3.79^c^	55.67 ± 4.38^a’b’^	1.47 ± 0.11^c^	1.62 ± 0.13^a’b’^	100.00 ± 0.00^a^	100.00 ± 0.00^a’^
4.0	20.89 ± 0.57^c^	21.83 ± 0.56^b’c’^	50.02 ± 5.11^c^	61.95 ± 3.10^b’c’^	1.46 ± 0.15^c^	1.80 ± 0.09^b’c’^	100.00 ± 0.00^a^	100.00 ± 0.00^a’^
6.0	20.95 ± 0.38^c^	22.42 ± 0.05^c’^	50.19 ± 5.54^c^	64.48 ± 4.80^c’^	1.46 ± 0.16^c^	1.88 ± 0.14^c’^	100.00 ± 0.00^a^	100.00 ± 0.00^a’^
8.0	19.90 ± 0.51^bc^	22.48 ± 0.35^c’^	47.06 ± 4.11^bc^	65.02 ± 6.03^c’^	1.37 ± 0.12^bc^	1.89 ± 0.18^c’^	100.00 ± 0.00^a^	100.00 ± 0.00^a’^
10.0	19.14 ± 0.74^ab^	22.44 ± 0.28^c’^	39.94 ± 4.20^ab^	64.54 ± 5.02^c’^	1.16 ± 0.12^ab^	1.88 ± 0.15^c’^	100.00 ± 0.00^a^	100.00 ± 0.00^a’^
12.0	18.57 ± 0.62^a^	22.51 ± 0.76^c’^	35.01 ± 2.51^a^	65.59 ± 5.29^c’^	1.02 ± 0.07^a^	1.91 ± 0.15^c’^	100.00 ± 0.00^a^	100.00 ± 0.00^a’^

*Note*: Values are mean ± SD of 3 replicates, with 20 fish in each replicate. Values in the same column with the different superscripts are significantly different (*p* < 0.05).

### 3.2. How Dietary LPE and HPE Effects on Body and Organ Parameters of Fish

**Figure 3:**
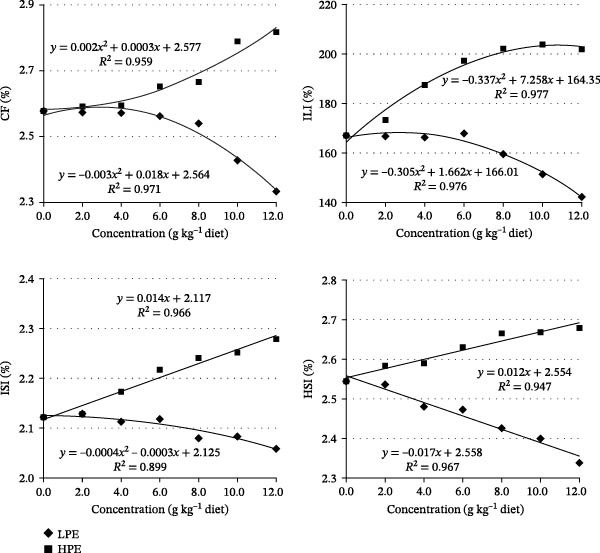
Linear regression analysis of condition factor (CF), intestinal length index (ILI), intestosomatic index (ISI), and hepatosomatic index (HSI) of Jian carp fed diets containing graded levels of low or high polarity extract of *Angelica sinensis* (LPE or HPE) for 42 days. Values are mean ± SD of 3 replicates, with 10 fish in each replicate.

As presented in Table 4, all measured parameters including body length, CF, intestinal length, ILI, intestinal weight, hepatopancreas weight, and HSI exhibited decreasing trends with increasing dietary LPE levels in Jian carp (*p* < 0.05, Figure 3). In contrast, body length, CF, intestinal length, ILI, intestine weight, ISI, hepatopancreas weight, and HSI were obviously increased in carp supplemented with the increases in HPE levels in the diets (*p* < 0.05, Figure 3). ISI was not significantly affected by varying levels of LPE supplementation (*p* > 0.05, Table 4).

**Table 4:** Body length, condition factor (CF), intestinal length, intestinal length index (ILI), intestine weight, intestosomatic index (ISI), hepatopancreas weight, and hepatosomatic index (HSI) of Jian carp fed diets containing graded levels of low or high polarity extract of *Angelica sinensis* (LPE or HPE) for 42 days.

Concentration (g kg^−1^ diet)	Body length (cm)		CF (%)		Intestinal length (cm)		ILI (%)	
	LPE	HPE	LPE	HPE	LPE	HPE	LPE	HPE
0.0	8.58 ± 0.16^ba’^		2.58 ± 0.14^ba’^		14.32 ± 0.8^ba’^		167.02 ± 11.19^ca’^	
2.0	8.59 ± 0.22^b^	8.59 ± 0.21^a’^	2.57 ± 0.20^b^	2.59 ± 0.19^a’^	14.31 ± 0.94^b^	14.87 ± 1.17^a’^	166.69 ± 11.88^c^	173.28 ± 15.29^a’^
4.0	8.59 ± 0.18^b^	8.71 ± 0.19^a’b’^	2.57 ± 0.16^b^	2.59 ± 0.17^a’^	14.28 ± 1.08^b^	16.32 ± 1.21^b’^	166.28 ± 12.78^c^	187.45 ± 14.24^b’^
6.0	8.52 ± 0.19^b^	8.77 ± 0.22^b’^	2.56 ± 0.18^b^	2.65 ± 0.20^a’b’^	14.30 ± 1.30^b^	17.28 ± 0.71^c’^	167.85 ± 14.95^c^	197.22 ± 10.94^b’c’^
8.0	8.45 ± 0.22^b^	8.80 ± 0.19^b’^	2.54 ± 0.19^b^	2.67 ± 0.18^a’b’^	13.47 ± 1.05^b^	17.78 ± 0.77^c’^	159.49 ± 12.84^bc^	202.12 ± 9.68^c’^
10.0	8.24 ± 0.17^a^	8.85 ± 0.16^b’^	2.43 ± 0.15^ab^	2.79 ± 0.16^b’^	12.47 ± 1.14^a^	18.03 ± 1.34^c’^	151.33 ± 13.63^ab^	203.77 ± 15.38^c’^
12.0	8.13 ± 0.19^a^	9.04 ± 0.20^c’^	2.33 ± 0.17^a^	2.82 ± 0.18^b’^	11.56 ± 1.07^a^	18.24 ± 0.75^c’^	142.22 ± 13.09^a^	201.87 ± 9.59^c’^

	**Intestine weight (g)**		**ISI (%)**		**Hepatopancreas weight (g)**		**HSI (%)**	
	**LPE**	**HPE**	**LPE**	**HPE**	**LPE**	**HPE**	**LPE**	**HPE**

0.0	0.34 ± 0.02^da’^		2.12 ± 0.11^aa’^		0.41 ± 0.02^ea’^		2.54 ± 0.1^ba’^	
2.0	0.35 ± 0.02^d^	0.35 ± 0.02^a’^	2.13 ± 0.15^a^	2.13 ± 0.11^a’^	0.41 ± 0.01^e^	0.42 ± 0.02^a’b’^	2.54 ± 0.13^b^	2.58 ± 0.15^a’b’^
4.0	0.34 ± 0.02^d^	0.37 ± 0.02^b’^	2.11 ± 0.11^a^	2.17 ± 0.15^a’b’^	0.40 ± 0.02^de^	0.44 ± 0.01^b’^	2.48 ± 0.19^ab^	2.59 ± 0.14^a’b’^
6.0	0.33 ± 0.01^cd^	0.40 ± 0.02^c’^	2.12 ± 0.12^a^	2.22 ± 0.12^a’b’^	0.39 ± 0.02^d^	0.47 ± 0.03^c’^	2.47 ± 0.14^ab^	2.63 ± 0.12^a’b’^
8.0	0.32 ± 0.02^c^	0.41 ± 0.02^c’^	2.08 ± 0.11^a^	2.24 ± 0.10^b’^	0.37 ± 0.02^c^	0.48 ± 0.03^c’^	2.43 ± 0.19^ab^	2.67 ± 0.11^b’^
10.0	0.28 ± 0.02^b^	0.44 ± 0.03^d’^	2.08 ± 0.09^a^	2.25 ± 0.08^b’^	0.32 ± 0.02^b^	0.52 ± 0.02^d’^	2.40 ± 0.14^ab^	2.67 ± 0.08^b’^
12.0	0.26 ± 0.02^a^	0.47 ± 0.02^e’^	2.06 ± 0.06^a^	2.28 ± 0.05^b’^	0.29 ± 0.02^a^	0.56 ± 0.02^e’^	2.34 ± 0.11^a^	2.68 ± 0.09^b’^

*Note*: Values are mean ± SD of 3 replicates, with 10 fish in each replicate. Values with the different superscripts in the same column are significantly different (*p* < 0.05).

### 3.3. How Dietary LPE and HPE Effects on Body Composition Parameters of Fish

**Figure 4:**
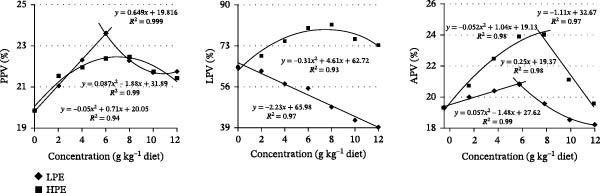
Linear regression analysis of protein, lipid, and ash productive value (PPV, LPV, and APV) of Jian carp fed diets containing graded levels of low or high polarity extract of *Angelica sinensis* (LPE or HPE) for 42 days. Values are mean ± SD of 3 replicates, with 5 fish in each replicate.

As presented in Table 5, neither BMC nor APV showed significant variations with increasing levels of LPE supplementation. Similarly, dietary HPE supplementation had no significant effects on BMC, BPC, and BAC. Compared to the control group, increasing dietary LPE levels significantly enhanced BPC and BAC (*p* < 0.05), while reducing BLC and LPV in Jian carp (*p* < 0.05, Table 5). PPV showed a trend of first increasing and then decreasing in carp with LPE supplementation, with the highest value observed in carp fed the diet containing 6.0 g LPE kg^−1^ (Figure 4). In contrast, increasing HPE supplementation significantly elevated BLC when contrasted with the control group (*p* < 0.05, Table 5). Moreover, PPV, LPV, and APV showed a trend of first increasing and then decreasing in carp with HPE supplementation (Figure 4). The optimum dietary HPE levels for PPV, LPV, and APV were 6.0, 8.0, and 8.0 g kg^−1^ supplementation in Jian carp, respectively (Figure 4).

**Table 5:** Body moisture, protein, lipid, and ash content (BMC, BPC, BLC, and BAC) as well as protein, lipid, and ash productive value (PPV, LPV, and APV) of Jian carp fed diets containing graded levels of low or high polarity extract of *Angelica sinensis* (LPE or HPE) for 42 days.

Concentration (g kg^−1^ diet)	BMC (%)		BPC (%)		BLC (%)		BAC (%)	
	LPE	HPE	LPE	HPE	LPE	HPE	LPE	HPE
0.0	76.69 ± 1.23^aa’^		13.62 ± 0.53^aa’^		5.76 ± 0.07^da’^		2.91 ± 0.11^aa’^	
2.0	76.40 ± 1.32^a^	76.62 ± 1.22^a’^	14.00 ± 0.89^ab^	13.61 ± 0.65^a’^	5.61 ± 0.27^cd^	5.77 ± 0.17^a’^	2.95 ± 0.22^ab^	2.89 ± 0.10^a’^
4.0	75.66 ± 1.74^a^	76.18 ± 1.07^a’^	14.93 ± 0.78^bc^	13.48 ± 0.45^a’^	5.41 ± 0.08^bc^	5.87 ± 0.26^a’b’^	3.06 ± 0.13^abc^	2.91 ± 0.09^a’^
6.0	75.50 ± 1.55^a^	75.70 ± 1.65^a’^	15.36 ± 0.16^cd^	13.36 ± 1.07^a’^	5.24 ± 0.21^ab^	5.94 ± 0.30^a’b’^	3.08 ± 0.01^abc^	2.92 ± 0.04^a’^
8.0	75.38 ± 1.48^a^	75.46 ± 1.72^a’^	15.82 ± 0.38^cde^	13.38 ± 1.09^a’^	5.07 ± 0.17^a^	6.00 ± 0.20^a’b’^	3.16 ± 0.12^bc^	2.93 ± 0.17^a’^
10.0	74.80 ± 1.38^a^	75.36 ± 1.36^a’^	16.33 ± 0.79^de^	13.33 ± 0.59^a’^	4.93 ± 0.20^a^	6.15 ± 0.12^a’b’^	3.19 ± 0.10^bc^	2.88 ± 0.12^a’^
12.0	74.21 ± 1.88^a^	75.17 ± 1.27^a’^	17.03 ± 1.04^e^	13.70 ± 0.74^a’^	4.94 ± 0.14^a^	6.25 ± 0.18^b’^	3.29 ± 0.15^c^	2.83 ± 0.17^a’^

	PPV (%)		LPV (%)		APV (%)	
	LPE	HPE	LPE	HPE	LPE	HPE
0.0	19.85 ± 1.02^aa’^		64.07 ± 0.56^ea’^		19.29 ± 0.88^aa’^	
2.0	21.08 ± 1.77^ab^	21.60 ± 0.92^a’b’^	62.52 ± 3.80^de^	68.84 ± 4.27^a’b’^	19.97 ± 1.88^a^	20.71 ± 0.56^a’b’^
4.0	22.38 ± 2.12^ab^	23.05 ± 1.00^b’c’^	57.23 ± 4.83^cd^	74.91 ± 2.61^b’c’^	20.36 ± 2.03^a^	22.45 ± 1.57^b’c’d’^
6.0	23.74 ± 1.97^b^	24.18 ± 1.52^c’^	55.36 ± 3.34^c^	80.40 ± 5.77^c’^	20.81 ± 1.97^a^	23.87 ± 2.25^c’d’^
8.0	22.37 ± 1.33^ab^	24.32 ± 0.77^c’^	47.59 ± 3.21^b^	81.79 ± 6.38^c’^	19.58 ± 1.16^a^	24.01 ± 1.81^d’^
10.0	21.79 ± 2.17^ab^	21.81 ± 1.79^a’b’^	42.11 ± 2.67^ab^	75.83 ± 3.14^b’c’^	18.53 ± 1.73^a^	21.10 ± 1.43^a’b’c’^
12.0	21.81 ± 1.89^ab^	21.49 ± 1.62^a’b’^	39.23 ± 2.28^a^	73.28 ± 6.55^b’c’^	18.22 ± 1.74^a^	19.56 ± 1.50^a’^

*Note*: Values are mean ± SD of 3 replicates, with 5 fish in each replicate. Values with the different superscripts in the same column are significantly different (*p* < 0.05).

### 3.4. How Dietary LPE and HPE Influences the Respiratory Metabolism in Fish

**Figure 5:**
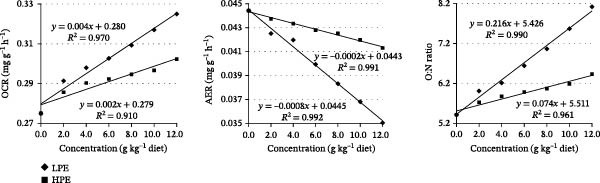
Linear regression analysis of oxygen consumption rate (OCR), ammonia excretion rate (AER), and O:N ratio in Jian carp fed diets containing graded levels of low or high polarity extract of *Angelica sinensis* (LPE or HPE) for 42 days. Values are mean ± SD of 3 replicates, with 5 fish in each replicate.

Relative to the control group, fish in the LPE‐treated groups exhibited significant lower FAC, FDO, and AER (*p* < 0.05), along with significant higher OCR and O:N ratio (*p* < 0.05, Table 6). The FDO was decreased, while OCR and O:N ratio were enhanced in HPE‐treated carp (*p* < 0.05, Table 6). Dietary HPE supplementation showed no significant effects on fish FAC and AER across the tested concentration ranges (Table 6). The metabolic index above gradually changed with the increase in LPE and HPE concentrations in diets (Figure 5). Notably, OCR and O:N ratio values in the LPE treatment groups were greater than those of HPE, while AER of LPE treatment groups was lower than those of HPE (Figure 5).

**Table 6:** Fish body weight (BW), water volume (WV), final dissolved oxygen (FDO), and final ammonia concentrations (FAC) in water and oxygen consumption rate (OCR), ammonia excretion rate (AER), and O:N ratio in Jian carp fed diets containing graded levels of low or high polarity extract of *Angelica sinensis* (LPE or HPE) for 42 days.

Concentration (g kg^−1^ diet)	BW (g fish^−1^)		WV (L bottle^−1^)		FDO (mg L^−1^)		FAC (μmol L^−1^)	
	LPE	HPE	LPE	HPE	LPE	HPE	LPE	HPE
0.0	16.68 ± 0.53^aa’^		4.17 ± 0.13^aa’^		5.48 ± 0.18^cb’^		77.27 ± 0.91^ea’^	
2.0	16.92 ± 0.51^a^	16.77 ± 0.59^a’^	4.23 ± 0.13^a^	4.19 ± 0.15^a’^	5.32 ± 0.12^bc^	5.37 ± 0.12^a’b’^	76.15 ± 1.80^de^	76.87 ± 1.68^a’^
4.0	16.78 ± 0.56^a^	16.63 ± 0.54^a’^	4.19 ± 0.14^a^	4.16 ± 0.14^a’^	5.25 ± 0.15^bc^	5.33 ± 0.14^a’b’^	75.83 ± 0.84^cde^	76.63 ± 1.32^a’^
6.0	16.88 ± 0.49^a^	16.93 ± 0.45^a’^	4.22 ± 0.12^a^	4.23 ± 0.11^a’^	5.20 ± 0.09^ab^	5.31 ± 0.10^a’b’^	74.63 ± 1.46^bcd^	76.31 ± 1.38^a’^
8.0	16.58 ± 0.52^a^	16.86 ± 0.73^a’^	4.14 ± 0.13^a^	4.22 ± 0.18^a’^	5.14 ± 0.11^ab^	5.28 ± 0.14^a’b’^	73.68 ± 0.91^abc^	76.15 ± 1.80^a’^
10.0	17.01 ± 0.65^a^	17.00 ± 0.38^a’^	4.25 ± 0.16^a^	4.25 ± 0.09^a’^	5.06 ± 0.11^ab^	5.26 ± 0.12^a’b’^	72.80 ± 1.70^ab^	75.83 ± 0.84^a’^
12.0	16.67 ± 0.70^a^	17.05 ± 0.53^a’^	4.17 ± 0.17^a^	4.26 ± 0.13^a’^	4.98 ± 0.17^a^	5.21 ± 0.10^a’^	71.76 ± 0.73^a^	75.43 ± 1.73^a’^

	OCR (mg g^−1^ h^−1^)		AER (mg g^−1^ h^−1^)		O:N ratio	
	LPE	HPE	LPE	HPE	LPE	HPE
0.0	0.275 ± 0.018^aa’^		0.044 ± 0.002^ea’^		5.42 ± 0.33^aa’^	
2.0	0.291 ± 0.012^ab^	0.286 ± 0.012^a’b’^	0.043 ± 0.003^de^	0.044 ± 0.003^a’^	6.01 ± 0.41^ab^	5.73 ± 0.33^a’b’^
4.0	0.298 ± 0.015^ab^	0.290 ± 0.014^a’b’^	0.042 ± 0.001^cde^	0.043 ± 0.002^a’^	6.21 ± 0.32^ab^	5.88 ± 0.50^a’b’^
6.0	0.303 ± 0.009^bc^	0.292 ± 0.010^a’b’^	0.040 ± 0.002^bcd^	0.043 ± 0.002^a’^	6.64 ± 0.36^bc^	5.99 ± 0.35^a’b’^
8.0	0.309 ± 0.011^bc^	0.295 ± 0.014^a’b’^	0.038 ± 0.002^abc^	0.043 ± 0.003^a’^	7.07 ± 0.13^cd^	6.07 ± 0.18^a’b’^
10.0	0.317 ± 0.011^bc^	0.297 ± 0.012^a’b’^	0.037 ± 0.003^ab^	0.042 ± 0.001^a’^	7.57 ± 0.75^de^	6.19 ± 0.37^b’^
12.0	0.325 ± 0.017^c^	0.302 ± 0.010^b’^	0.035 ± 0.001^a^	0.041 ± 0.003^a’^	8.12 ± 0.54^e^	6.43 ± 0.59^b’^

*Note*: IDO = 8.23 ± 0.21 mg L^−1^; IAC = 51.14 ± 3.15 μmol L^−1^; DT = 0.50 h. Values are means ± S.D. of 3 replicates, with 5 fish in each replicate. Values in the same column with the different superscripts are significantly different (*p* < 0.05).

Abbreviations: DT, durative time; IAC, initial ammonia concentration; IDO, initial dissolved oxygen.

### 3.5. How Dietary LPE and HPE Influences the Biochemical Parameters in Plasma of Fish

In comparison with control group, GOT and LDH activity as well as PA and TAA level exhibited a marked decrease, TP showed an apparently increase in the plasma of LPE and HPE treatment groups (*p* < 0.05, Table 7). Nonetheless, dietary LPE and HPE enhanced and reduced GLU level in the plasma of Jian carp, respectively (*p* < 0.05, Table 7). TG level was decreased in the plasma of Jian carp of LPE supplementation (*p* < 0.05, Table 7). No significant changes in serum TG levels were observed in response to varying levels of dietary HPE supplementation (Table 7).

**Table 7:** The activities of glutamate‐oxaloacetate transaminase (GOT) and lactate dehydrogenase (LDH) as well as the content of plasma mmonia (PA), total amino acids (TAAs), protein (TP), glucose (GLU), and triglyceride (TG) in plasma of Jian carp fed diets containing graded levels of low or high polarity extract of *Angelica sinensis* (LPE or HPE) for 42 days.

Concentration (g kg^−1^ diet)	GOT (U L^−1^)		LDH (U L^−1^)		PA (μmol L^−1^)		TAA (µmoL mL^−1^)	
	LPE	HPE	LPE	HPE	LPE	HPE	LPE	HPE
0.0	104.9 ± 9.46^ed’^		882.07 ± 56.41^dd’^		496.74 ± 26.55^ec’^		39.82 ± 2.87^ed’^	
2.0	97.64 ± 7.92^de^	80.84 ± 6.34^c’^	826.53 ± 46.21^cd^	695.13 ± 57.18^c’^	458.60 ± 18.92^d^	483.26 ± 31.90^b’c’^	37.50 ± 2.60^de^	36.07 ± 2.33^c’^
4.0	91.58 ± 6.15^cd^	67.31 ± 6.04^b’^	805.80 ± 60.24^c^	618.03 ± 57.26^b’^	431.63 ± 20.71^cd^	482.33 ± 49.43^b’c’^	36.07 ± 2.24^cd^	34.64 ± 2.31^b’c’^
6.0	83.33 ± 7.34^bc^	59.53 ± 6.27^a’b’^	723.73 ± 67.21^b^	573.26 ± 53.36^a’b’^	402.79 ± 26.79^c^	466.98 ± 38.68^b’c’^	35.18 ± 3.26^cd^	33.21 ± 2.63^b’c’^
8.0	78.25 ± 7.43^ab^	60.45 ± 5.72^a’b’^	650.78 ± 59.20^a^	567.88 ± 40.32^a’b’^	366.51 ± 13.01^b^	437.21 ± 26.72^b’^	33.57 ± 1.96^bc^	31.61 ± 2.80^a’b’^
10.0	70.79 ± 6.17^a^	55.17 ± 2.84^a’^	626.28 ± 52.97^a^	559.59 ± 50.85^a’b’^	338.14 ± 24.59^ab^	383.26 ± 18.34^a’^	31.25 ± 1.89^ab^	29.64 ± 2.22^a’^
12.0	70.52 ± 4.56^a^	54.09 ± 3.05^a’^	581.55 ± 42.24^a^	504.87 ± 26.32^a’^	321.86 ± 27.98^a^	365.58 ± 41.58^a’^	29.46 ± 2.75^a^	28.39 ± 2.40^a’^

	TP (mg mL^−1^)		GLU (mmol L^−1^)		TG (mmol L^−1^)	
	LPE	HPE	LPE	HPE	LPE	HPE
0.0	17.25 ± 1.26^aa’^		10.03 ± 0.47^ad’^		0.55 ± 0.04^ea’^	
2.0	18.05 ± 1.74^ab^	20.45 ± 1.83^b’^	10.11 ± 0.46^a^	9.32 ± 0.59^c’^	0.52 ± 0.02^de^	0.53 ± 0.03^a’^
4.0	19.51 ± 1.82^abc^	22.54 ± 1.64^b’c’^	10.56 ± 0.51^ab^	8.71 ± 0.26^b’^	0.50 ± 0.02^cd^	0.54 ± 0.05^a’^
6.0	20.24 ± 1.84^bc^	24.52 ± 2.07^c’^	11.12 ± 0.59^bc^	8.46 ± 0.19^a’b’^	0.48 ± 0.04^bcd^	0.55 ± 0.05^a’^
8.0	20.96 ± 1.75^c^	24.97 ± 2.24^c’^	11.38 ± 0.81^bcd^	8.42 ± 0.18^a’b’^	0.47 ± 0.03^abc^	0.56 ± 0.05^a’^
10.0	21.90 ± 2.09^c^	24.54 ± 1.28^c’^	11.81 ± 0.83^cd^	8.16 ± 0.19^a’^	0.45 ± 0.02^ab^	0.56 ± 0.04^a’^
12.0	22.07 ± 2.01^c^	24.01 ± 2.31^c’^	12.09 ± 0.40^d^	8.21 ± 0.22^a’^	0.43 ± 0.03^a^	0.58 ± 0.05^a’^

*Note*: Values are mean ± SD of 3 replicates, with 5 fish in each replicate. Values with the different superscripts in the same column are significantly different (*p* < 0.05).

### 3.6. How Dietary LPE and HPE Influences the Biochemical Index in Intestines of Fish

In comparison with the control, the activity of α‐amylase in intestine increased progressively with higher levels of dietary LPE supplementation in diets of Jian carp (*p* < 0.05, Table 8). The activities of trypsin and AKP first increased and then decreased in intestines of LPE‐treated carp, reaching peak values at 6.0 and 8.0 g LPE kg^−1^ of diet, respectively (*p* < 0.05, Table 8). Lipase, Na^+^, K^+^‐ATPase, and γ‐GT activities showed a trend of initial decline followed by a increase in intestines of carp treated with LPE (*p* < 0.05), reached the lowest values in 6.0, 8.0, and 8.0 g LPE kg diet^−1^, respectively (Table 8).

**Table 8:** The activities of trypsin, lipase, α‐amylase, Na^+^, K^+^‐ATPase, alkaline phosphatase (AKP), and gamma‐glutamyl transpeptidase (γ‐GT) in intestines of Jian carp fed diets containing graded levels of low or high polarity extract of *Angelica sinensis* (LPE or HPE) for 42 days.

Concentration (g kg^−1^ diet)	Trypsin (U mg protein^−1^)		Lipase (U g protein^−1^)		α‐Amylase (U mg protein^−1^)	
	LPE	HPE	LPE	HPE	LPE	HPE
0.0	670.81 ± 140.60^ba’^		25.53 ± 1.24^ba’^		0.87 ± 0.02^aa’^	
2.0	678.87 ± 128.83^b^	781.80 ± 156.67^a’b’^	24.79 ± 2.09^ab^	28.38 ± 2.49^a’b’^	0.94 ± 0.08^b^	0.85 ± 0.05^a’^
4.0	680.67 ± 131.58^b^	785.26 ± 108.27^a’b’^	23.75 ± 2.42^ab^	29.32 ± 2.28^b’^	0.98 ± 0.03^bc^	0.84 ± 0.04^a’^
6.0	705.34 ± 38.81^b^	823.32 ± 150.36^a’b’c’^	22.53 ± 1.23^a^	29.07 ± 2.13^b’^	1.02 ± 0.04^cd^	0.83 ± 0.06^a’^
8.0	659.94 ± 122.38^b^	839.25 ± 78.05^a’b’c’^	23.01 ± 1.38^a^	30.28 ± 1.95^b’^	1.04 ± 0.04^cde^	0.84 ± 0.03^a’^
10.0	443.09 ± 61.20^a^	845.72 ± 56.18^b’c’^	23.30 ± 1.74^ab^	30.88 ± 2.80^b’^	1.06 ± 0.07^de^	0.83 ± 0.04^a’^
12.0	377.81 ± 40.40^a^	936.70 ± 108.71^c’^	23.44 ± 1.50^ab^	30.31 ± 2.65^b’^	1.09 ± 0.02^e^	0.83 ± 0.04^a’^

	**Na^+^,K^+^-ATPase (U mg protein^−1^)**		**AKP (U g protein^−1^)**		**γ-GT (U g protein^−1^)**	
	**LPE**	**HPE**	**LPE**	**HPE**	**LPE**	**HPE**

0.0	3.57 ± 0.30^ba’^		157.56 ± 8.21^aa’^		4.46 ± 0.29^ba’^	
2.0	3.48 ± 0.27^ab^	3.78 ± 0.27^a’b’^	173.37 ± 11.99^ab^	189.76 ± 16.44^b’^	4.29 ± 0.34^ab^	4.75 ± 0.28^a’b’^
4.0	3.37 ± 0.14^ab^	3.90 ± 0.26^a’b’c’^	180.95 ± 12.35^bc^	199.74 ± 15.69^b’c’^	4.13 ± 0.38^ab^	4.82 ± 0.37^a’b’^
6.0	3.27 ± 0.32^ab^	4.08 ± 0.29^b’c’^	190.69 ± 15.81^bc^	210.74 ± 16.21^b’c’^	3.94 ± 0.33^a^	5.01 ± 0.39^b’^
8.0	3.18 ± 0.21^a^	4.20 ± 0.27^c’^	197.08 ± 19.05^c^	216.53 ± 19.41^c’^	3.87 ± 0.22^a^	4.98 ± 0.17^b’^
10.0	3.26 ± 0.24^ab^	4.24 ± 0.22^c’^	196.25 ± 14.74^c^	211.51 ± 6.59^c’^	3.97 ± 0.21^a^	4.95 ± 0.33^b’^
12.0	3.33 ± 0.24^ab^	4.10 ± 0.34^b’c’^	189.39 ± 11.63^bc^	207.66 ± 17.29^b’c’^	4.13 ± 0.37^ab^	4.83 ± 0.34^a’b’^

*Note*: Values are mean ± SD of 3 replicates, with 5 fish in each replicate. Values with the different superscripts in the same column are significantly different (*p* < 0.05).

In contrast to the control, dietary HPE supplementation significantly enhanced intestinal trypsin and lipase activities in Jian carp, which gradually increased with the increase in HPE concentrations in diets (*p* < 0.05, Table 8). The activities of Na^+^, K^+^‐ATPase, AKP, and γ‐GT showed a trend of first increasing and then decreasing in intestines of HPE–treated carp (*p* < 0.05), reached the highest values in 10.0, 8.0, and 6.0 g HPE kg diet^−1^, respectively (Table 8). Oral administration of HPE did not significantly affect intestinal α‐amylase activity in Jian carp (*p* > 0.05, Table 8).

### 3.7. How Dietary LPE or HPE Influences the Biochemical Index in Hepatopancreas of Fish

In contrast to the control, hepatopancreatic trypsin and lipase activities in LPE–treated Jian carp exhibited biphasic responses, showing initial increases followed by decreases (*p* < 0.05, Table 9). Maximal enzyme activities were observed at dietary LPE levels of 4.0 g kg^−1^ (trypsin) and 2.0 g kg^−1^ (lipase), whereas significant reductions occurred at higher concentrations of 10.0 and 8.0 g kg^−1^, respectively (Table 9). Additionally, the activities of α‐amylase, GOT, and GPT in hepatopancreas demonstrated dose‐dependent increases with rising dietary LPE concentrations (*p* < 0.05, Table 9). Oral LPE administration showed no significant effect on LDH activity in the hepatopancreas of Jian carp.

**Table 9:** The activities of trypsin, lipase, α‐amylase, glutamate‐oxaloacetate transaminase (GOT), glutamate‐pyruvate transaminase (GPT), and lactate dehydrogenase (LDH) in hepatopancreas of Jian carp fed diets containing graded levels of low or high polarity extract of *Angelica sinensis* (LPE or HPE) for 42 days.

Concentration (g kg^−1^ diet)	Trypsin (U mg protein^−1^)		Lipase (U g protein^−1^)		α‐Amylase (U mg protein^−1^)	
	LPE	HPE	LPE	HPE	LPE	HPE
0.0	1453.17 ± 90.63^aba’^		42.54 ± 3.57^cda’^		0.97 ± 0.05^aa’^	
2.0	1514.92 ± 112.79^b^	1477.82 ± 100.36^a’b’^	45.85 ± 2.05^d^	43.94 ± 2.22^a’b’^	1.00 ± 0.06^ab^	0.96 ± 0.05^a’^
4.0	1543.06 ± 110.69^b^	1504.35 ± 121.11^a’b’^	45.73 ± 2.15^d^	44.95 ± 4.31^a’b’^	1.01 ± 0.05^ab^	0.95 ± 0.02^a’^
6.0	1529.75 ± 125.67^b^	1545.82 ± 99.59^a’b’^	43.89 ± 3.72^cd^	48.50 ± 3.35^b’c’^	1.02 ± 0.03^ab^	0.94 ± 0.05^a’^
8.0	1474.20 ± 129.00^b^	1566.25 ± 60.83^a’b’^	41.00 ± 3.21^c^	49.67 ± 4.29^c’^	1.04 ± 0.04^b^	0.93 ± 0.04^a’^
10.0	1401.64 ± 123.35^ab^	1579.08 ± 78.17^a’b’^	36.91 ± 1.32^b^	53.13 ± 2.90^c’^	1.04 ± 0.05^b^	0.93 ± 0.03^a’^
12.0	1308.76 ± 120.35^a^	1615.64 ± 138.12^b’^	32.16 ± 2.48^a^	59.37 ± 2.80^d’^	1.05 ± 0.05^b^	0.95 ± 0.03^a’^

	**GOT (U g protein^−1^)**		**GPT (U g protein^−1^)**		**LDH (U g protein^−1^)**	
	**LPE**	**HPE**	**LPE**	**HPE**	**LPE**	**HPE**

0.0	22.44 ± 1.86^aa’^		62.44 ± 3.84^aa’^		109.23 ± 6.28^aa’^	
2.0	27.46 ± 1.97^b^	23.03 ± 2.21^a’^	67.03 ± 2.92^ab^	62.30 ± 5.86^a’^	113.67 ± 5.44^a^	110.55 ± 6.02^a’^
4.0	29.66 ± 1.01^b^	23.19 ± 2.08^a’^	71.27 ± 6.36^bc^	62.76 ± 5.19^a’^	113.98 ± 8.18^a^	114.13 ± 7.36^a’b’^
6.0	33.76 ± 1.00^c^	24.04 ± 1.74^a’^	78.08 ± 7.78^c^	63.01 ± 5.67^a’^	114.13 ± 7.65^a^	119.84 ± 11.26^a’b’c’^
8.0	34.59 ± 2.26^c^	23.97 ± 2.18^a’^	92.10 ± 7.75^d^	64.34 ± 4.55^a’^	115.91 ± 9.48^a^	124.07 ± 10.37^b’c’^
10.0	35.95 ± 2.12^cd^	24.35 ± 2.39^a’^	99.06 ± 1.98^d^	64.42 ± 6.36^a’^	115.56 ± 9.63^a^	130.56 ± 7.55^c’d’^
12.0	37.60 ± 1.71^d^	25.14 ± 2.03^a’^	112.81 ± 9.74^e^	65.03 ± 5.94^a’^	116.96 ± 11.70^a^	136.01 ± 10.78^d’^

*Note*: Values are mean ± SD of 3 replicates, with 5 fish in each replicate. Values with the different superscripts in the same column are significantly different (*p* < 0.05).

As compared with the control, hepatopancreatic activities of trypsin, lipase and LDH in Jian carp showed dose‐dependent increases with rising dietary HPE concentrations (*p* < 0.05, Table 9). In contrast, α‐amylase, GOT, and GPT activities remained unaffected by varying HPE supplementation levels (Table 9).

## 4. Discussion

Plasma GOT and LDH activities serve as primary sensitive biomarkers for hepatocyte damage [31]. Hepatic tissue injury triggers the release of GOT and LDH into circulation, elevating their plasma concentrations [32, 33]. The current investigation showed that dietary LPE and HPE supplementation significantly reduced plasma GOT and LDH activities in carp. The hepatoprotective effects of LPE and HPE on fish are likely attributable to their major bioactive constituents—ligustilide and polysaccharides, respectively, which have been pharmacologically identified as core active components of AS [9]. In present research, LPE and HPE contained up to 26.69% of ligustilide and 50.84% of polysaccharides, respectively. Consistent with our findings, Luo et al. [34] reported that ligustilide suppressed serum LDH activity in bleomycin‐induced rats, while Wang et al. [35] observed that AS polysaccharides decreased serum GOT activity in concanavalin A‐challenged mice. Our results confirm the nontoxic nature of LPE and HPE in fish.

### 4.1. Regulatory Effects of Dietary LPE on Growth, Digestion, Absorption, Body Composition, and Metabolism in Fish

In fish, FE, WG, and SGR can reflect the growth performance [36, 37]. The current findings indicated that carps fed diets supplemented with LPE reduced FE, WG, and SGR, aligning with earlier observations of AS suppressed body WG in obese mice [10]. Broken‐line regression analysis indicated that WG at 6.18 g kg^−1^ inclusion level of LPE in diet showed a steep decrease, suggesting that is the maximum addition level of LPE required to maintain fish WG. Growth performance was adversely influenced by the presence of LPE in the diet, according to the current results. Different from the above research, previous studies showed that dietary MPE improves the growth performance in carp [23]. The difference in growth may be ascribed to their chemical compositions. Studies demonstrated that MPE contains more ferulic acid and less ligustilide than LPE [17]. AS was found to exert no significant enhancement in the growth performance of yellow catfish [8]. Nevertheless, ferulic acid improved the growth performance in carp and tilapia [14]. So, it is likely that ligustilide had an inhibitory effect on the growth of fish. However, the correlation between ligustilide and fish growth performance remains insufficiently explored, necessitating further experiments to elucidate its regulatory mechanisms.

FI shows significant positive correlation with growth performance in fish [38]. The current experiment demonstrated that LPE‐supplemented diet significantly reduced FI in common carp. One possible explanation for this alteration is the underdevelopment of the digestive system in juvenile crap [16]. As vital digestive organs in fish, hepatopancreas and intestine play crucial roles in nutrient digestion and absorption [39]. Our data revealed significant reductions in hepatopancreatic weight, HSI, intestinal weight, intestinal length, and ILI in LPE–fed carp, suggesting that dietary LPE may inhibit the development of digestive organs in juvenile fish. Currently, there is no available literature addressing how LPE affects fish digestive organs. In fish, FI is largely dependent on the efficiency of digestive and absorptive enzyme systems, as these enzymes facilitate the breakdown of nutrients and directly help improve appetite and nutrient utilization [33]. Among them, trypsin plays a vital role by catalyzing the hydrolysis of proteins and polypeptides into amino acids and small peptides [40]. Lipase mediates the hydrolysis of ester bonds in substrates including TGs, phospholipids, and cholesteryl esters [41]. α‐Amylase hydrolyzes starch into GLU and maltose [42]. Analysis of the results revealed that dietary LPE decreased lipase activity while increasing α‐amylase activity in both hepatopancreas and intestine of carp. A similar trend was also documented by Li et al. [16] regarding MPL–induced enhancement of α‐amylase activity in the digestive system of carp.

Na^+^, K^+^‐ATPase mediates transmembrane transport of phosphate, amino acids and GLU in animals [43]. AKP not only promotes the breakdown of phosphate compounds, resulting in the release of phosphate ions and alkaline products, but also facilitates nutrient absorption (including GLU, lipids, inorganic phosphate, and calcium) in fish intestine [44, 45]. γ‐GT exhibits dual functions: (1) mediating gamma‐glutamyl transfer to amino acid receptors (critical for cellular amino acid absorption) and (2) catalyzing glutathione cleavage into glutamic acid, cysteine, and glycine [46]. Our investigation revealed that dietary LPE decreased intestinal Na^+^, K^+^‐ATPase and γ‐GT activities, while increasing AKP activity in common carp. These findings align with report on MEP‐induced enhancement of intestinal AKP activity in carp [16]. Currently, literature regarding LPE’s effects on digestive and absorptive enzymes in fish hepatopancreas and intestine remains limited. The collective data suggest that dietary LPE may modulate nutritional utilization in fish by suppressing protein and lipid digestive and absorptive capacities while enhancing that of carbohydrates by fish. This conclusion is substantiated by plasma biochemical profiles that after 42 days of LPE supplementation, significant reductions in TAA and TG concomitant with elevated GLU levels were observed in carp plasma.

It has been indicated that ligustilide decreases the levels of TG in serum and lipid accumulation in hepatocytes of mice with dyslipidemia [47]. So, it is possible that LPE affected the body composition of fish. In harmony with the hypothesis above, in the research, dietary LPE decreased CF, body lipid level, and LPV and increased body protein and ash levels as well as PPV in carp. This result is consistent with the reports that AS volatile oil (low polarity component) can reduce the content of TG and free fatty acid in the liver of high‐fat diet mice [48] and has a certain lipid‐lowering effect on hyperlipidemic rats [49]. Moreover, studies have displayed that ligustilide reduces obesity index (the calculation formula is the same as CF) in diabetes rats [11] and the accumulation of fat in skeletal muscle by regulating cytokines related to GLU and lipid metabolism in high‐fat diet mice [50]. These results demonstrated that dietary LPE supplements administration assists to cut down lipid accumulation and promote protein accumulation in fish body.

The effects of LPE on the body composition may have a close association with the energy metabolism in fish. The processes of respiration and excretion are central to the maintenance of energy homeostasis in animals [51]. In fish physiology, OCR is considered as a reliable metric for evaluating aerobic metabolic rate and energy status [22]. Our findings showed that dietary LPE enhanced OCR in carp, indicating intensified aerobic energy metabolism. This result is not in accordance with the report that dietary MPE containing ferulic acid decreased OCR in carp under hypoxia [17]. However, dietary ferulic acid increases OCR in the Cu‐exposed carp [14]. The reasons for the differences above may be due to the different components in LPE and MPE and the interference caused by stress factors on the fish body as stated above. Since protein is the primary energy source [27], ammonia acts as the major end product of the protein catabolism in teleosts, the increase in ammonia excretion reflects elevated protein catabolism [52]. In the present study, dietary LPE declined AER in carp. These results indicated that LPE enhances the catabolism of nutrition, but reduces the catabolism of protein in fish. O:N ratio can provide the information on changes in energy substrate (nutrients) utilization by animals [51]. An increase in O:N ratio indicates an elevation in lipid and carbohydrate metabolisms [51]. The present result suggested that dietary LPE enhanced O:N ratio in the metabolism of carp. To date, there is a lack of published data regarding the effects of LPE on the aforementioned indicators in fish. These results confirmed that dietary LPE enhances the catabolism of fat and carbohydrates, while reducing the catabolism of proteins in fish body.

Fish primarily derive energy from protein, and optimizing the dietary protein‐to‐energy ratio can attenuate protein catabolism [53]. As Eliason et al. [54] demonstrated, protein represents the most costly macronutrient in fish feeds, with excess amounts being catabolized for energy production. Protein metabolism in fish is predominantly regulated by two aminotransferases (GOT and GPT), their forward reactions channel amino acids into the TCA cycle for energy generation via deamination [55, 56], whereas reverse reactions facilitate amino acid anabolism [57, 58]. The hepatopancreas of fish is not only an important digestive organ but also an important metabolic organ [59]. The changes in metabolic enzyme activity in liver can reflect the energy supply status of animals [60]. Our findings revealed that dietary LPE supplementation enhanced hepatopancreatic GOT and GPT activities while reducing PA content in Jian carp. This result was in good agreement with the report that MPE increased the activities of GOT and GPT in hepatopancreas and decreased the level of ammonia in plasma in red carp [16]. PER serves as a crucial indicator for evaluating protein utilization efficiency in fish [61, 62], and plasma TP levels reflect systemic protein metabolism [63]. Intriguingly, LPE supplementation decreased PER values but significantly elevated plasma TP levels in carp. Collectively, these results suggest that LPE may modulate protein metabolism by suppressing proteolysis while promoting synthesis and deposition of protein.

### 4.2. Effects of Dietary HPE on Growth Performance, Digestion, Absorption, Body Composition, and Metabolism in Fish

Wang et al. demonstrated that orally administered AS polysaccharides can be absorbed throughout the entire small intestine in mice [64]. In the present study, HPE was found to contain 19.55% crude protein, 20.24% reducing sugars, and 50.84% polysaccharides. Following ingestion, AS polysaccharides progress through the digestive tract, migrating with intestinal chyme to the distal intestinal region, where they underwent enzymatic degradation into lower molecular weight fractions that are subsequently utilized in the murine distal intestine [65]. These findings suggest that fish may benefit from appropriate supplementation of HPE by digestion and absorption. In this study, dietary HPE supplements for 42 days raised FE, WG, and SGR in carp. This is in line with the reports that supplementation of AS water extraction and alcohol precipitation containing polysaccharides for 42 days improves WG in chicks [66], oral administration of AS polysaccharides for 28 days increases the BW of diabetes rats [67]. Broken‐line regression analysis based on WG indicated that the optimal supplementation level was 5.91 g HPE kg^−1^ of diet. These results demonstrated that dietary HPE can increase fish growth performance.

Fish growth is positively correlated with digestive and absorptive capacity [38]. The present study demonstrated that dietary HPE supplementation significantly enhanced FI, intestinal length, ILI, intestinal weight, ISI, hepatopancreas weight, and HSI in carp. To our knowledge, no prior studies have investigated the effects of HPE on the digestive organs of fish. These findings suggest that dietary HPE promotes the development of digestive organs in juvenile fish. Digestive enzymes, including trypsin, lipase, and α‐amylase in the hepatopancreas and intestine, play a crucial role in nutrient breakdown, and their activity serves as a reliable indicator of digestive efficiency in fish [68]. In this study, dietary HPE significantly increased trypsin and lipase activities in both the hepatopancreas and intestine of carp. Intestinal AKP, Na^+^, K^+^‐ATPase, and γ‐GT are key enzymes involved in nutrient absorption, and their activity levels reflect the absorptive capacity of fish [69]. Our results revealed that dietary HPE supplementation markedly elevated AKP, Na^+^, K^+^‐ATPase, and γ‐GT activities in the intestine of carp. Currently, limited information exists regarding the effects of HPE on digestive and absorptive enzyme activities in the hepatopancreas and intestine of fish. The data presented above indicate that dietary HPE enhances the digestive and absorptive capacities for protein and fat in fish, suggesting its potential to optimize nutrient utilization.

The impact of HPE on digestive and absorptive capacities may be closely linked to the body composition of fish. Supporting the above hypothesis, in this research, dietary HPE increased CF, lipid content, PPV, and LPV in carp body. This result is consistent with the report that polysaccharides from AS enhance CF and lipid content in golden pompano (but not significant) [19] and from *Lycium barbarum* increase the protein content in muscles of juvenile golden pompano [70]. These results demonstrate that dietary HPE supplements administration assists to promote lipid accumulation in fish body. The observed alterations in fish body composition may be attributed to modifications in metabolic regulation. In this study, dietary HPE increased OCR and O:N ratio in carp metabolism. There are no published studies on the effects of HPE on the above indicators in fish. These results indicate that dietary HPE enhances the catabolism of carbohydrates and fat in fish. Supporting the above conclusion, in this study, dietary HPE significantly reduced GLU level, but increased TG level in plasma of carp (not significant). This result is in accordance with the report that GLU levels in plasma are reduced after a 4‐week oral administration of AS polysaccharides in diabetic mice [71]. Different from the above result, AS polysaccharides reduce the level of TG in plasma of diabetic rats [72]. The reason for the result about TG in plasma may be the increase in lipase and absorptive enzyme activity in digestive organs of fish caused by HPE as stated above. Moreover, in this study, dietary HPE increased PPV in carp, indicating an increase in protein accumulation in fish. The reason for the above results may be related to protein metabolism in fish. In this study, dietary HPE supplements significantly decreased the levels of PA in plasma as well as the activities of GPT and GOT in hepatopancreas of carp. This result was consistent with the previous report about oral high‐dose HPE in Jian carp [12]. The results above confirm that dietary HPE reduces the catabolism of amino acid in fish. In this study, dietary HPE decreased the level of TAA, but increased the level of TP in plasma of carp. This is in good harmony with the report that dietary AS polysaccharides enhanced TP content in plasma of golden pompano (but not significant) [19]. These findings suggest that incorporating HPE into the diet promotes protein anabolism in fish, highlighting its potential for optimizing growth and metabolism.

Feed‐derived carbohydrates not only function as important precursors in the biosynthesis of lipids and nonessential amino acids, but also efficient carbohydrate utilization supplies energy for growth and physiological functions, thereby enhancing the efficiency of feed protein utilization in animals [73]. GLU levels in plasma can be used to assess glycometabolism in fish [74, 75]. The results of this study align with previous findings that dietary AS polysaccharides reduced GLU content in plasma of golden pompano, although the changes were not statistically significant [19]. LDH is a critical enzyme in anaerobic glycolysis, aiding in energy generation through GLU metabolism [76]. In the research, dietary HPE supplementation led to an increase in LDH activity in the hepatopancreas of carp. This result was in line with the report that dietary MPE increased in LDH activity in red blood cells of carp [17]. The current findings suggest that including HPE into the diet can boost the energy supply of GLU metabolism in fish.

## 5. Conclusion

In summary, dietary LPE reduces the growth and accumulation of body lipid and enhances the accumulation of body proteins by decreasing the digestion and absorption of lipids as well as amino acid catabolism and increasing the catabolism of sugar and fat in fish. At the same time, the results confirm that dietary HPE improves the growth performance and accumulation of body lipid and proteins by decreasing the catabolism of amino acid and increasing the digestion and absorption of proteins and lipids and the catabolism of sugar in fish. The optimal supplementation levels of LPE and HPE in diets for fish growth are 6.18 and 5.91 g kg^−1^, respectively. This study reveals the reasons why AS affects the growth of fish and provides a basis for using its extract as a functional raw material for fish feed.

## Funding

This work was supported by the Sichuan Science and Technology Program (Grant 2025ZNSFSC0206) and the Talent Program (Grant R2019015) of Neijiang Normal University.

## Conflicts of Interest

The authors declare no conflicts of interest.

## Data Availability

The data of this study can be provided by the corresponding author upon request.
